# Delayed Hemodynamic Collapse From Subclavian Artery Perforation Following Transradial Coronary Intervention, Requiring Rescue Endovascular Stenting

**DOI:** 10.7759/cureus.117903

**Published:** 2026-10-14

**Authors:** Amaan Khan, Montasir Elmobark, Neil Swanson, Ahmed Abdelatie, Danish Ahmed

**Affiliations:** 1 Internal Medicine/Cardiology, James Cook University Hospital, Middlesbrough, GBR; 2 Acute Medicine, University Hospital of North Tees, Stockton-on-Tees, GBR; 3 Cardiology, James Cook University Hospital, Middlesbrough, GBR

**Keywords:** bleeding risk, ct optimization, interventional cardiologist, intraoperative complication, subclavian artery perforation, surgical techniques

## Abstract

We report a case of a 53-year-old man who developed proximal right subclavian artery perforation during coronary angiography and percutaneous coronary intervention, leading to a large mediastinal hematoma and resultant delayed hemodynamic collapse. Although initial angiography demonstrated a proximal subclavian artery dissection, subtle contrast extravasation was only identified on retrospective review. Subsequent hemodynamic deterioration prompted computed tomography angiography, which demonstrated active arterial extravasation and a large mediastinal hematoma requiring emergency intervention.

## Introduction

Transradial access is widely used for coronary angiography and percutaneous coronary intervention (PCI) because of its favorable bleeding and vascular complication profile compared with femoral access. However, although uncommon, injury to the proximal arterial vasculature, including the subclavian and brachiocephalic arteries, can result in life-threatening mediastinal hemorrhage. Recognition may be challenging when initial hemodynamic parameters are preserved, and angiographic evidence of vascular injury is subtle. This case demonstrates delayed hemodynamic collapse following an initially unrecognized proximal subclavian artery perforation and highlights the importance of persistent shoulder or chest pain following difficult catheter passage through tortuous subclavian anatomy [1].

## Case presentation

A 53-year-old man presented with a non-ST-elevation myocardial infarction. His background was significant for diffuse, complex coronary artery disease requiring multiple previous PCIs. Prior to admission, he had experienced progressive angina despite medical therapy, with known residual disease involving the left circumflex and right coronary arteries. Initial chest radiography on admission demonstrated no evidence of mediastinal widening or pleural abnormality (Figure 1).

**Figure 1:**
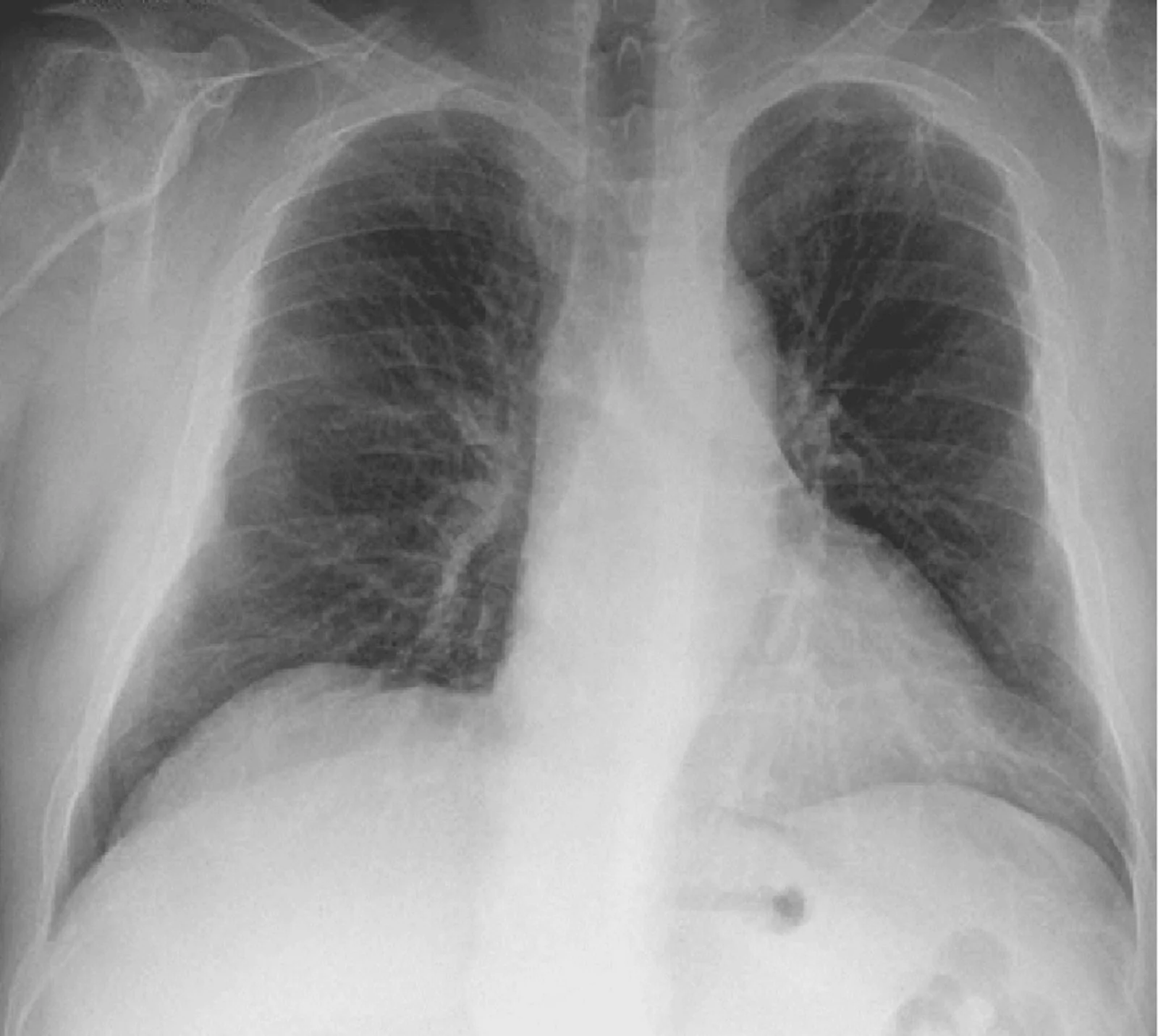
Initial chest radiograph on admission demonstrating no evidence of mediastinal widening or pleural abnormality, providing an important baseline before the development of thoracic vascular injury

Diagnostic coronary angiography was performed via the right radial artery using a 6-Fr Terumo Glidesheath Slender (Terumo Medical Corporation, Elkton, MD). Angiography demonstrated severe in-stent restenosis within the left circumflex artery, which was considered the culprit lesion for his presentation. The procedure was complicated by marked tortuosity of the right subclavian artery, resulting in significant difficulty advancing the guide catheter. Multiple attempts were made to advance an Extra Backup 4.0 guide catheter beyond the subclavian tortuosity; however, this was unsuccessful, and an alternative access strategy was pursued.

A 0.038-inch guidewire was used during catheter advancement. There was no hydrophilic guidewire use or documented forceful guidewire manipulation; however, catheter advancement remained technically challenging.

An attempt at left radial artery access was unsuccessful, and the procedure was subsequently completed via right femoral artery access. The left coronary system was successfully engaged using a 6-Fr Judkins Left 4.0 guide catheter, and the obtuse marginal branches were wired using standard coronary guidewires.

During coronary intervention, the patient developed new right shoulder discomfort. There were no ECG changes during this time, and the patient remained hemodynamically stable with a heart rate of 80 beats/minute and a systolic blood pressure of 131 mmHg. In the context of an acute coronary syndrome and ongoing intervention, this was initially felt to represent ongoing ischemic symptoms related to the left circumflex lesion. The lesion was subsequently treated with angioplasty and stenting with a satisfactory angiographic result (Figure 2).

**Figure 2:**
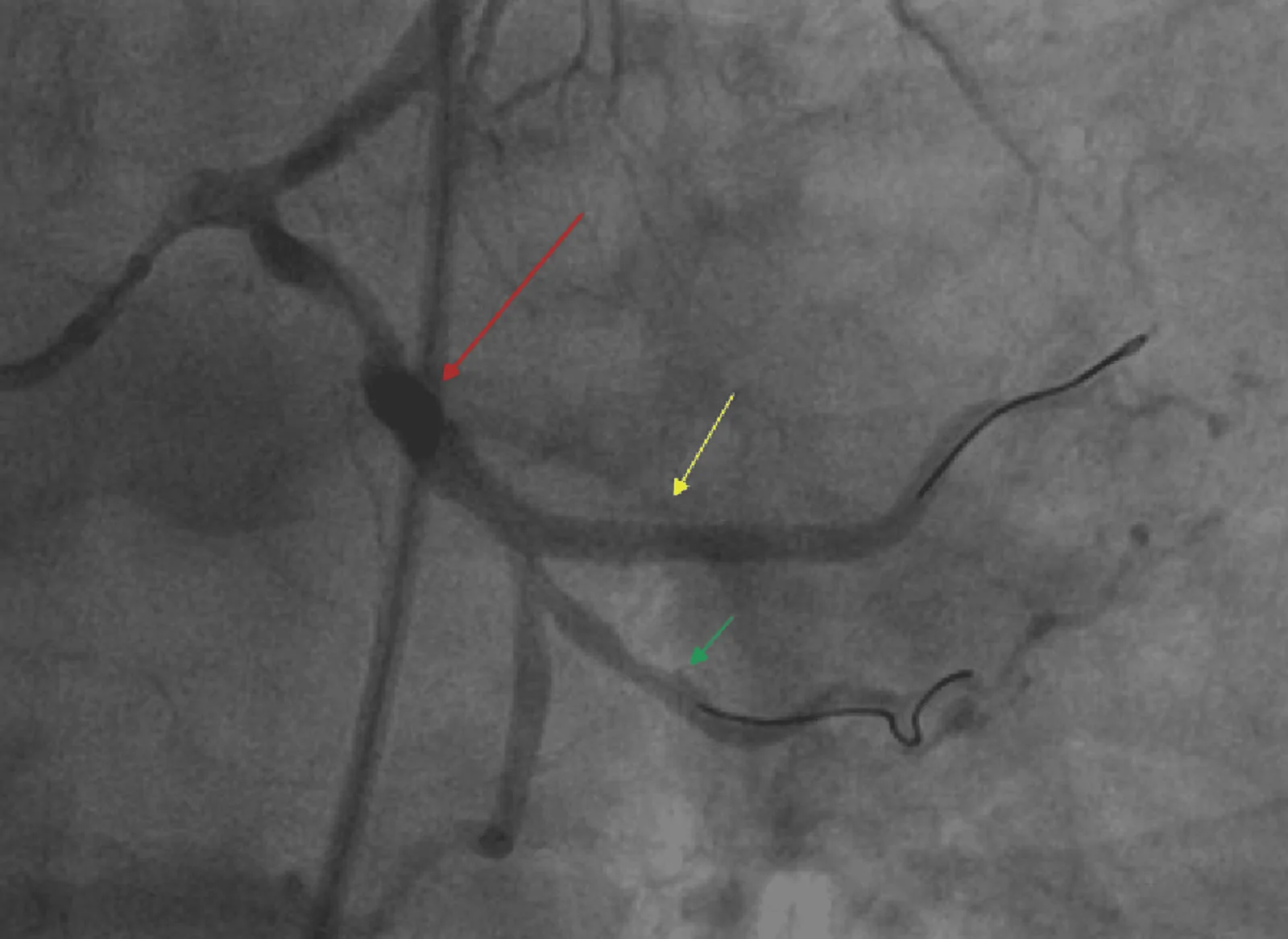
Final coronary angiogram demonstrating successful percutaneous coronary intervention to obtuse marginal branch extending into the left circumflex artery The red arrow shows the circumflex artery, the yellow arrow shows the OM1 branch, and the green arrow shows the OM2 branch OM1: first obtuse marginal; OM2: second obtuse marginal

Assessing symptoms was also challenging because the patient’s first language was not English, limiting detailed communication about the character and evolution of his discomfort. This may have contributed to the initial attribution of his symptoms to ongoing myocardial ischemia rather than evolving vascular injury.

Following successful treatment of the coronary lesion, the patient reported persistent chest and shoulder pain. In view of the ongoing symptoms and technically challenging radial access, angiography of the proximal right subclavian artery was performed, demonstrating a proximal subclavian artery dissection (Figure 3).

**Figure 3:**
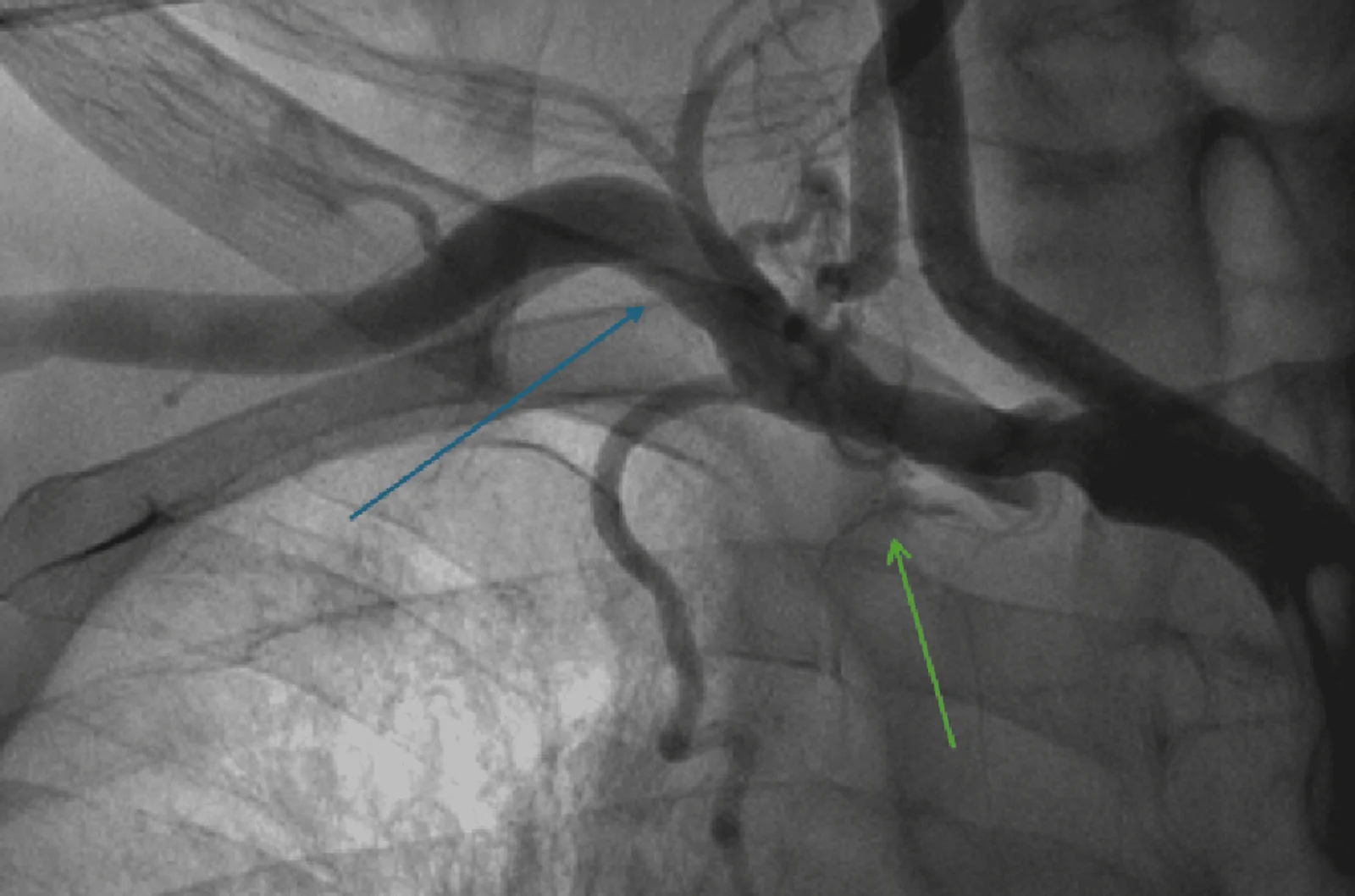
Initial angiography of the proximal right subclavian artery demonstrating dissection with subtle contrast extravasation at the site of vascular injury. The extravasation was not recognized during the initial angiographic assessment but was identified on retrospective review The blue arrow shows the subclavian artery, and the green arrow shows the subtle contrast extravasation

Although subtle contrast extravasation was present on retrospective review, this was not recognized during the initial angiographic assessment, when the predominant finding was interpreted as arterial dissection. As the patient remained hemodynamically stable with no ECG changes, an initial conservative strategy with close monitoring in the coronary care unit was adopted. Shortly after transfer to the coronary care unit, the patient developed sudden hemodynamic deterioration with profound hypotension and circulatory collapse. Emergency assessment was undertaken with input from vascular surgery and cardiothoracic surgery. Bedside transthoracic echocardiography excluded pericardial effusion, raising concern for an alternative source of bleeding. Repeat chest radiography demonstrated new marked mediastinal widening (Figure 4).

**Figure 4:**
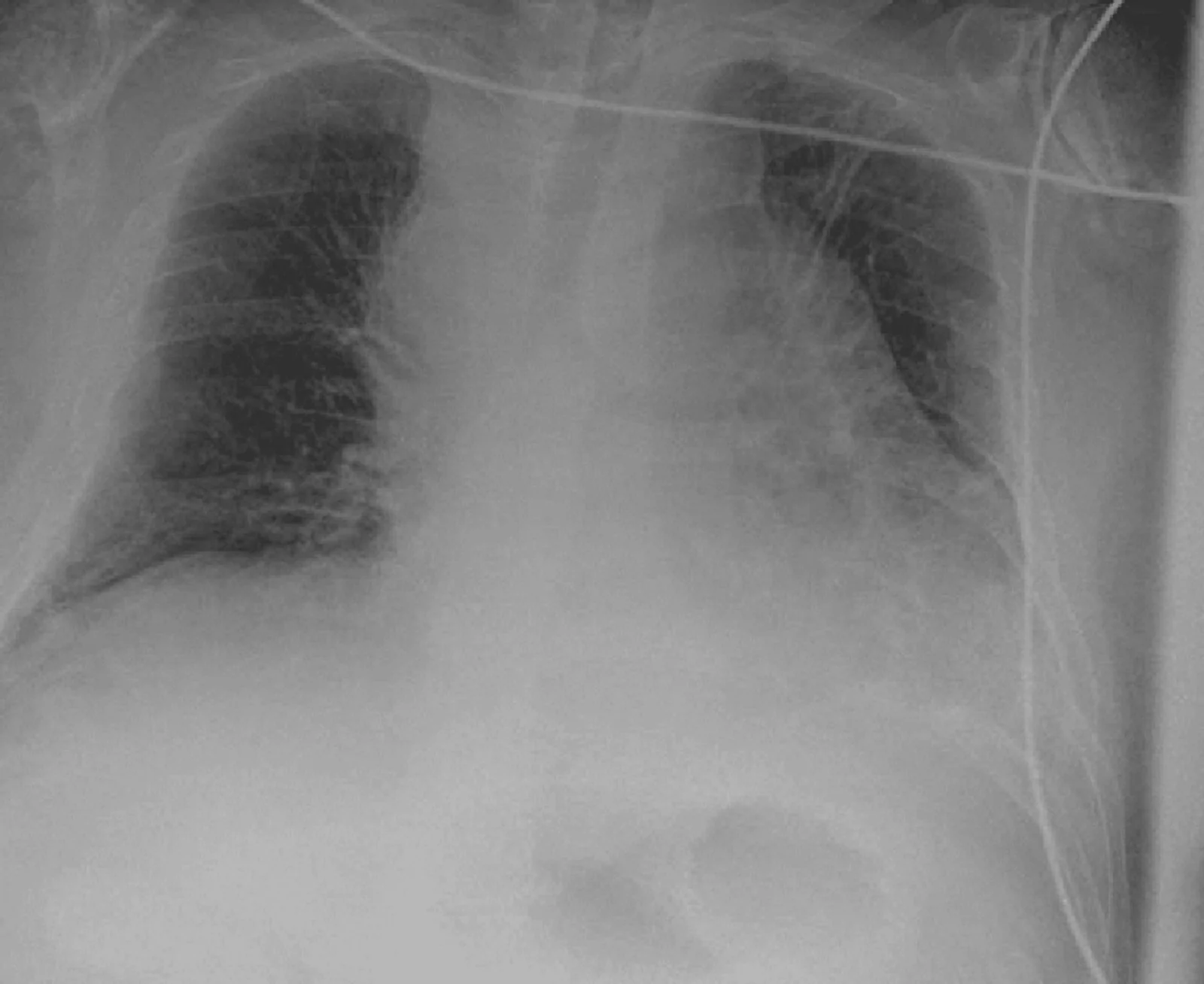
Chest radiograph following hemodynamic deterioration demonstrating marked mediastinal widening, consistent with the development of a large mediastinal hematoma secondary to proximal subclavian artery perforation

Urgent CT angiography of the aorta demonstrated a large mediastinal hematoma with active contrast extravasation arising from the origin of the right subclavian artery, immediately distal to the brachiocephalic artery bifurcation, associated with a small dissection flap (Figure 5).

**Figure 5:**
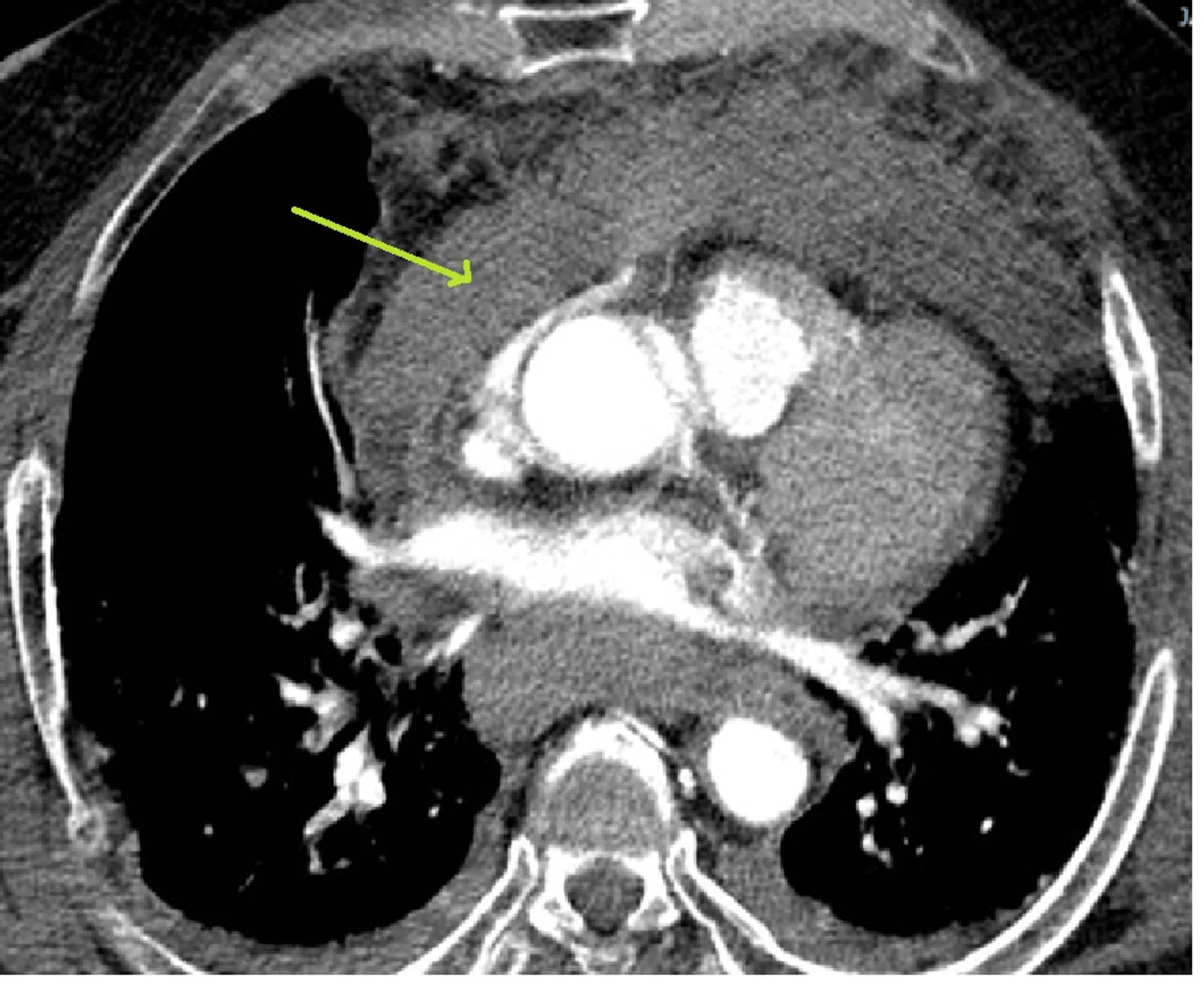
Contrast-enhanced computed tomography angiography demonstrating active contrast extravasation arising from the proximal right subclavian artery immediately distal to the brachiocephalic artery bifurcation associated with a large mediastinal hematoma. These findings prompted emergency multidisciplinary endovascular repair The green arrow shows extensive mediastinal hematoma

A multidisciplinary discussion involving interventional cardiology, vascular surgery, interventional radiology and cardiothoracic surgery was undertaken. The injury was considered anatomically challenging because of its proximity to the brachiocephalic artery and right common carotid artery origin. Given the patient's hemodynamic instability and the high mortality associated with ongoing mediastinal hemorrhage, emergency endovascular repair was deemed the most appropriate management strategy despite the recognized risk of cerebrovascular complications.

Surgical exposure of the right brachial artery was performed by the vascular surgery team, allowing percutaneous access by interventional radiology using micropuncture technique. The access sheath was subsequently upsized to facilitate endovascular intervention. The proximal right subclavian artery dissection was successfully crossed intraluminally, and a covered stent graft (10 mm × 5 cm Gore Viabahn, W. L. Gore & Associates, Flagstaff, AZ) was deployed across the site of injury. The covered stent was selected according to the diameter of the subclavian artery, with the aim of achieving adequate exclusion of the perforation while maintaining vessel patency. Given the subsequent hemodynamic deterioration and evidence of ongoing vascular injury, covered stent deployment was considered the most appropriate definitive endovascular strategy.

Following stent deployment, there was minor (<30%) coverage (“shuttering”) of the origin of the right common carotid artery; however, the vessel remained patent with preserved flow. Completion angiography demonstrated successful exclusion of the subclavian artery injury with no further active extravasation (Figure 6). The patient remained clinically stable following the intervention. Repeat computed tomography (CT) angiography is planned to assess the subclavian artery and confirm continued patency of the covered stent.

**Figure 6:**
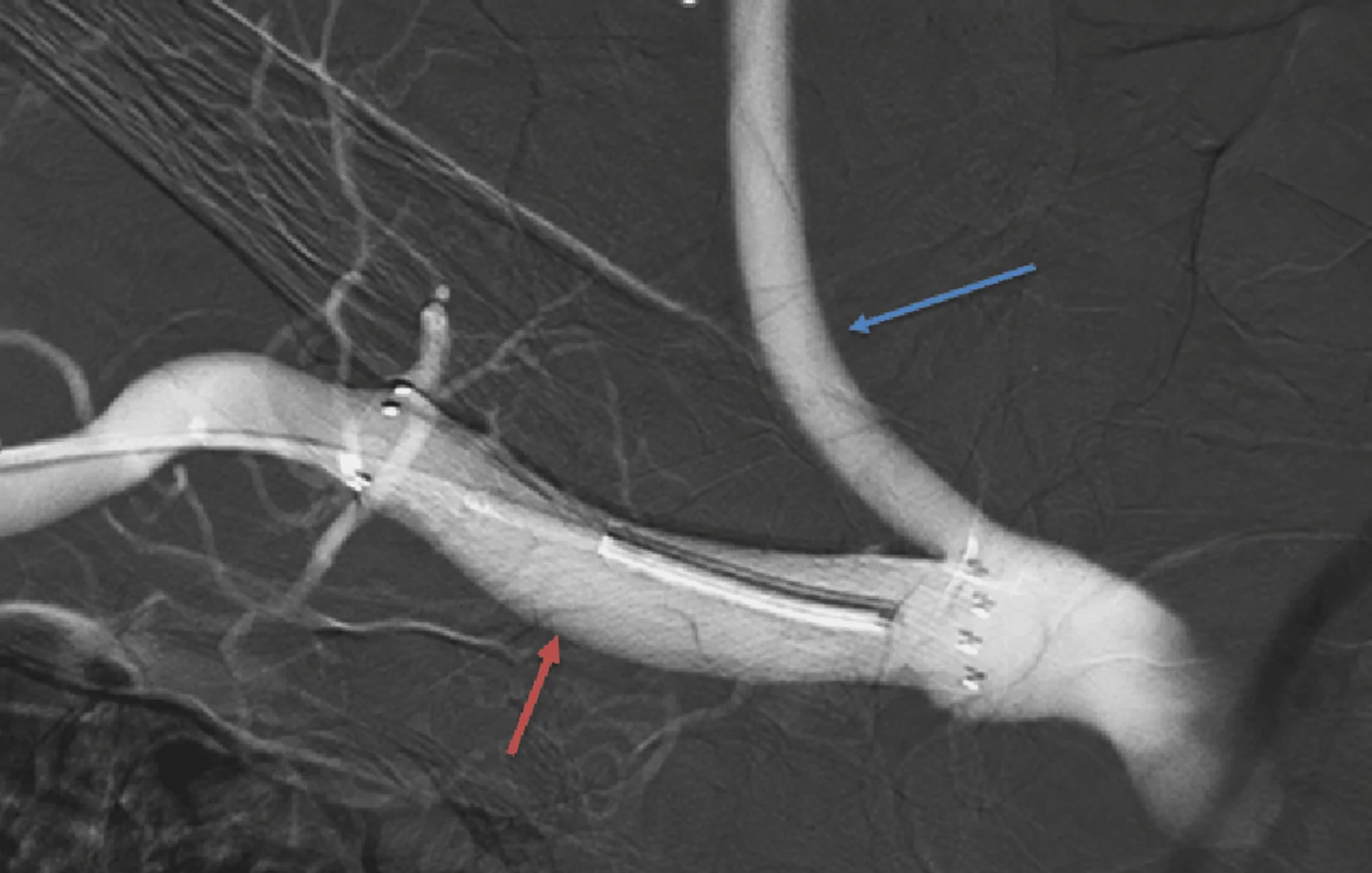
Completion angiography following deployment of a 10 mm × 50 mm Gore Viabahn-covered stent demonstrating complete exclusion of the proximal subclavian artery perforation with preservation of flow into the right common carotid and distal subclavian arteries The red arrow shows the right subclavian artery sealed after perforation, and the blue arrow shows the right common carotid artery

Serial laboratory investigations demonstrated a progressive decline in hemoglobin concentration (Table 1).

**Table 1:** Lab results

Measurement	Result	Reference
Hemoglobin	147 (on admission) -> 107 -> 87	130-180 g/L
Platelets	208 -> 251 -> 249	150-400 × 10^9^/L
C-reactive protein	<5 -> 81 -> 96	<5 mg/L
High-sensitivity troponin I	17 -> 31	<3 ng/L

Investigations

Table 2 summarizes the investigations performed and their principal findings.

**Table 2:** Investigations

Investigation	Findings
Initial chest radiograph	No evidence of mediastinal widening or pleural abnormality, providing a normal baseline prior to the subsequent vascular complication
Coronary angiography	Severe in-stent restenosis of the left circumflex artery, considered the culprit lesion for the non-ST elevation myocardial infarction. Marked tortuosity of the right subclavian artery was encountered during right radial catheter passage
Right subclavian angiography	Performed because of persistent chest and right shoulder pain following difficult radial access. This demonstrated a proximal right subclavian artery dissection. Retrospective review subsequently demonstrated subtle contrast extravasation at the site of vascular injury
Bedside transthoracic echocardiography	No evidence of pericardial effusion, raising suspicion of an extracardiac source of hemorrhage
Repeat chest radiograph	Marked mediastinal widening, consistent with the development of a large mediastinal hematoma
Urgent computed tomography angiography	Large mediastinal hematoma with active contrast extravasation from the proximal right subclavian artery, immediately distal to the brachiocephalic artery bifurcation, with an associated small dissection flap

Table 3 summarizes the differential diagnoses considered after the patient's hemodynamic deterioration.

**Table 3:** Differential diagnoses NSTEMI: non-ST-elevation myocardial infarction; PCI: percutaneous coronary intervention

S. no.	Differential diagnosis
1	Ongoing myocardial ischemia or an acute coronary complication, particularly given the patient's presentation with NSTEMI and development of symptoms during PCI
2	Coronary artery perforation with pericardial hemorrhage and cardiac tamponade, an important consideration following sudden hemodynamic deterioration after PCI; this was subsequently made unlikely by the absence of pericardial effusion on bedside echocardiography
3	Major access-related hemorrhage, including femoral or retroperitoneal bleeding, given that the coronary intervention was ultimately completed via femoral access
4	Proximal subclavian or brachiocephalic arterial injury, including dissection or perforation with mediastinal hemorrhage, particularly in view of the difficult catheter passage through the markedly tortuous right subclavian artery
5	Iatrogenic aortic injury or dissection, considered as an alternative vascular cause of acute chest pain and hemodynamic deterioration following catheter intervention

Outcome and follow-up

Following successful endovascular exclusion of the proximal right subclavian artery injury, the patient was transferred to the intensive care unit for ongoing monitoring and hemodynamic support. His circulatory status stabilized following the intervention, with no further clinical or radiological evidence of ongoing hemorrhage. His hemoglobin concentration declined from 147 g/L on admission to a nadir of 87 g/L, and he received three units of packed red blood cells, following which his hemoglobin stabilized.

Neurological assessment following the procedure revealed no evidence of cerebrovascular complications, despite the close proximity of the stent graft to the origin of the right common carotid artery. Follow-up vascular imaging confirmed patency of the covered stent, successful exclusion of the subclavian artery perforation, and no further contrast extravasation.

The patient made a satisfactory recovery and was subsequently discharged home. At cardiology outpatient review one month later, he remained clinically well, with no evidence of recurrent vascular complications. He was subsequently scheduled for routine follow-up with the cardiology nursing and cardiac rehabilitation teams. Table 4 summarizes the key learning points from this case.

**Table 4:** Learning points CT: computed tomography

S. no.	Learning points
1	Subclavian artery perforation is a rare but potentially life-threatening complication of transradial coronary intervention. A high index of suspicion should be maintained following difficult catheter manipulation, tortuous subclavian anatomy, or persistent shoulder/chest pain during or after the procedure
2	Persistent pain following coronary intervention should not automatically be attributed to ongoing myocardial ischemia. In the absence of pericardial effusion or significant ECG changes, extracardiac bleeding, including mediastinal hemorrhage, should be considered
3	Endovascular covered stent placement, supported by early multidisciplinary collaboration, can provide successful treatment for anatomically challenging proximal subclavian artery perforation
4	Subtle angiographic evidence of vascular injury may be missed. Careful review of angiographic images and early CT angiography when clinical findings are discordant can facilitate prompt diagnosis and management

## Discussion

In coronary angiography, radial access is now preferred over femoral access due to a significant reduction in complications with a transradial approach. In spite of this, deep vascular injuries within the thoracic inlet (including brachiocephalic and subclavian arteries), although rare, are underrecognized and associated with high morbidity and mortality [1].

The incidence of mediastinal hematoma has been shown to occur in under 0.01% of cases, with most evidence being derived from small case series or isolated case reports. Cases involve perforation of the subclavian artery or its branches [2].

Published reports identify several anatomical and procedural factors that may predispose to subclavian arterial injury during transradial coronary intervention. Vascular tortuosity, atherosclerotic disease, calcification, and acute vessel angulation may increase susceptibility to intimal injury during catheter passage [3]. In our case, marked subclavian tortuosity was encountered, resulting in technically challenging catheter advancement. Hydrophilic guidewires were not used within the subclavian artery, and there was no aggressive catheter manipulation. We therefore hypothesize that the severe tortuosity represented a major predisposing factor, with mechanical interaction between the catheter and the angulated vessel wall potentially resulting in dissection and subsequent perforation.

Although subclavian arterial injury has been associated with hydrophilic guidewires and forceful manipulation, neither was documented in this case. Vascular injury may nevertheless occur during technically challenging transradial procedures through interaction between the guidewire or catheter and the arterial wall, particularly in the presence of tortuous or unfavorable vascular anatomy. Repeated catheter advancement or contact with the vessel wall may result in intimal injury, with subsequent dissection and, rarely, perforation. Significant proximal vascular injury should therefore remain a consideration even in the absence of an obvious traumatic procedural event [2].

Patients may present with sudden hemodynamic instability, a drop in hemoglobin, and mediastinal widening on chest imaging. Signs including dyspnea, hypotension, and neck swelling have been described in the literature. Crucially, the absence of pericardial effusion and significant ECG changes should prompt consideration of extracardiac bleeding [3].

For definitive diagnosis of subclavian artery perforation, early CT angiography is recommended and facilitates identification of the bleeding source and allows planning of the potential endovascular intervention [3]. In our case, retrospective review of the subclavian angiogram demonstrated subtle contrast extravasation arising from the dissected segment, which was not recognized during the procedure. This highlights the importance of maintaining clinical suspicion and reassessing the imaging findings when the patient’s clinical course is inconsistent with the initial diagnosis.

In hemodynamically stable patients without active extravasation, successful conservative management has been reported. In the presence of active bleeding or hemodynamic instability, endovascular treatment provides a less invasive means of achieving hemostasis, with options including covered stent placement or selective embolization depending on the location and anatomy of the vascular injury. Surgical repair may be required when endovascular treatment is not technically feasible or fails to achieve hemostasis [4]. Where intervention is required, endovascular management using covered stents provides a minimally invasive means of excluding the site of arterial injury while preserving vessel patency and has been associated with high rates of technical success in reported series [5].

An important learning point from this case is understanding why the vascular injury was not recognized despite the patient developing new shoulder pain during the procedure. Several cognitive and clinical factors contributed to the delayed diagnosis. As the patient was undergoing PCI, ongoing myocardial ischemia was thought to be the most likely explanation for his shoulder pain. This represents a classic example of diagnostic anchoring, whereby subsequent clinical information was interpreted through the lens of the initial working diagnosis. Additionally, given the patient’s first language was not English, this made communication challenging, impairing more detailed assessments of his symptoms. Furthermore, the initial hemodynamic stability and the absence of readily apparent contrast extravasation on contemporaneous angiographic assessment reduced suspicion of a major vascular injury and supported the initial conservative strategy.

## Conclusions

This case presented a diagnostic challenge due to the lack of cardiac complications and access-site bleeding, further highlighting the importance of extracardiac bleeding as a source of injury. This case highlights the importance of early multidisciplinary collaboration between interventional cardiology, vascular surgery, interventional radiology, and cardiothoracic surgery in managing complex thoracic vascular complications. Rapid recognition and endovascular intervention allowed successful control of hemorrhage despite the challenging anatomical location of the injury.

Although exceedingly uncommon, proximal subclavian artery perforation should be considered whenever persistent shoulder or chest pain occurs following difficult transradial catheter manipulation.

Subclavian artery perforation is a rare but potentially life-threatening complication of transradial coronary intervention. A high index of suspicion should be maintained following difficult catheter manipulation, tortuous subclavian anatomy, or persistent shoulder/chest pain during or after the procedure. Persistent pain following coronary intervention should not automatically be attributed to ongoing myocardial ischemia. In the absence of pericardial effusion or significant ECG changes, extracardiac bleeding, including mediastinal hemorrhage, should be considered. Subtle angiographic evidence of vascular injury may be missed. Careful review of angiographic images and early CT angiography when clinical findings are discordant can facilitate prompt diagnosis and management. Endovascular covered stent placement, supported by early multidisciplinary collaboration, can provide successful treatment for anatomically challenging proximal subclavian artery perforation.
